# A Remedy for Crime? A Systematic Review on the Effects of Pharmacological ADHD Treatment on Criminal Recidivism and Rehabilitation in Inmates With ADHD

**DOI:** 10.1002/brb3.70120

**Published:** 2024-11-07

**Authors:** A. Carlander, M. Rydell, H. Kataoka, M. Hildebrand Karlén, A.‐S. Lindqvist Bagge

**Affiliations:** ^1^ SOM Institute University of Gothenburg Gothenburg Sweden; ^2^ Wallenberg Centre for Molecular and Translational Medicine University of Gothenburg Gothenburg Sweden; ^3^ Bra Liv Hälsan 2 Primary Health Care Centre Region Jönköping County Sweden; ^4^ Department of Psychology University of Gothenburg Gothenburg Sweden; ^5^ Department of Psychiatry and Neurochemistry, Institute of Neuroscience and Physiology, Sahlgrenska Academy University of Gothenburg Gothenburg Sweden

**Keywords:** ADHD, criminal recidivism, methylphenidate

## Abstract

**Introduction:**

There is a high prevalence of attention‐deficit/hyperactivity disorder (ADHD) in prison populations compared to the general population, and ADHD has also been shown to be associated with criminality and antisocial behavior. This systematic review examines the effect of pharmacological ADHD treatment on criminal recidivism, ADHD symptoms, and rehabilitation in inmates with ADHD.

**Methods:**

Adhering to PRISMA 2020 and AMSTAR guidelines, we conducted a structured search on September 6, 2023 using PubMed. We focused on original research published in peer‐reviewed scientific journals, following the IMRaD format, written in English, containing the established search terms, based on participants who met the criteria for ADHD diagnosis (any edition of DSM), and who were incarcerated at the start of pharmacological treatment for ADHD. The primary outcome was criminal recidivism, the secondary outcomes were ADHD symptoms, and rehabilitation‐related factors such as global function, norm‐breaking/antisocial behavior, adaptation to society/institutional behavior, cognitive function, and well‐being.

**Results:**

Five studies, based on three patient cohorts, were included in this systematic review. Surprisingly, only one study investigated criminal recidivism. That study indicated that self‐reported criminal recidivism was lower than expected among inmates who had received pharmacological ADHD treatment. The five studies showed varying results in the effectiveness of pharmacological ADHD treatment on ADHD symptoms and other rehabilitation‐related factors. The included studies also varied regarding participant characteristics, study design, dosage, adherence to treatment, treatment regimes, and measured outcomes. All studies reported using osmotic‐release oral system (OROS) methylphenidate as their drug of choice.

**Conclusion:**

We conclude that there is limited empirical evidence to support the efficacy of pharmacological ADHD treatment on criminal recidivism in inmates diagnosed with ADHD. Still, evidence suggests that these treatments can reduce ADHD symptoms and enhance rehabilitation outcomes, which may, in turn, lower the rate of reoffending. We point to the need for more targeted research in this area.

## Graphical abstract

ADHD is more common among inmates compared with the general population.

We perform a systematic review investigating if ADHD medication (methylphenidate) could reduce crime.

We find limited evidence but ADHD medication may facilitate rehabilitation and thereby reduce crime. More research is needed.

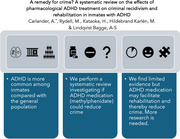

## 1 Introduction

Attention‐deficit/hyperactivity disorder (ADHD) is a neurodevelopmental disorder with childhood onset that is characterized by patterns of inattention and/or hyperactivity–impulsivity that interfere with daily life functioning (American Psychiatric Association 2013). These patterns often persist into adulthood and may be associated with increased risks of educational and occupational failure, antisocial behavior, and criminality (Asherson et al. 2016). Specifically, adults diagnosed with ADHD in childhood have a two to three times higher likelihood of arrests, convictions, incarceration, and criminal recidivism (Mohr‐Jensen and Steinhausen 2016). Research indicates that the prevalence of ADHD among prison inmates is about 26% (Baggio et al. 2018; Young et al. 2015), which is notably higher than the 2–3% often reported for the general adult population (Fayyad et al. 2017).

Pharmacological ADHD treatment has been reported to be effective in reducing ADHD symptoms such as inattention, impulsivity, hyperactivity (Faraone et al. 2021), emotional lability (Moukhtarian et al. 2017), as well as improving aspects of mental health, antisocial behavior, and academic and social function (Bihlar Muld et al. 2015).

The finding that pharmacological ADHD treatment is associated with a reduction in criminal activity has been reported in the World Federation of ADHD International Consensus Statement (Faraone et al. 2021). Additionally, it has been suggested that pharmacological ADHD treatment reduces criminal recidivism by mitigating the ADHD symptoms, thus aiding inmates and offenders to benefit to a higher degree from rehabilitation programs (Young et al. 2018).

Adults with ADHD often suffer from other mental health disorders, including anxiety disorders, mood disorders, substance use disorder (SUD), and various behavior disorders (Fayyad et al. 2017). A meta‐analysis has shown that inmates with ADHD (i.e., persons who met diagnostic/screening criteria for ADHD using validated measures), in comparison with inmates without ADHD, exhibit a higher incidence of behavioral problems and substance use but also antisocial personality disorder (ASPD) (Young et al. 2015). Specifically, it is common among inmates with ADHD to display a higher incidence of “reactive violence”, which is a spontaneous reaction in certain situations that triggers high affect, even when controlling for other factors such as ASPD and SUD (Retz and Rosler 2010). Furthermore, inmates with ADHD are reported to be involved more frequently in severe aggressive incidents during imprisonment (Young et al. 2009), and they also tend to exhibit an earlier onset of crime, more reported incidents of violence, as well as relapsing into crime more often and faster after release compared to other inmates (Retz et al. 2021).

There have been concerns about the validity of ADHD diagnoses and the effectiveness of pharmacological ADHD treatment in prison populations (Asherson et al. 2023). There are few published studies on the efficacy of such treatments in prison populations, for which high levels of comorbidity and substance use disorder may obscure the effectiveness of treatment. It has been posited that ADHD symptoms in inmates may instead stem from comorbidities like complex post‐traumatic stress disorder, personality disorders, substance use disorders, traumatic brain injuries, or other developmental disorders, where treatments with stimulants could prove ineffective, as discussed by Asherson et al. (2023).

A large research study utilizing national register data on 25,656 patients found that pharmacological treatment for ADHD had a significant inverse association with criminal behavior (Lichtenstein et al. 2012). Similarly, a registry study including released inmates reported a 42% lower rate of violent reoffending during periods when they were medicated with antipsychotics, psychostimulants, and drugs for addictive disorders (Chang et al. 2016). At the same time, they found no significant associations between violent reoffending and usage of antidepressants or antiepileptics.

Overall, the connection between ADHD and criminality is complex. As reviewed above, there is a high prevalence of ADHD among prison populations. Research has often reported an association indicating that pharmacological ADHD treatment is related to a lower degree of criminal behavior and criminal recidivism. It is, however, hard to establish a causal link between the use of pharmacological ADHD treatment among inmates and a reduction in criminal behavior and repeated offenses. To the best of our knowledge, there has been no review explicitly evaluating the effects of pharmacological treatment for ADHD on criminal recidivism and rehabilitation efforts among inmates diagnosed with ADHD.

### 1.1 Aim

The general aim of this systematic review is to identify and summarize the results of studies investigating the effects of recommended pharmacological treatment for ADHD in persons diagnosed with ADHD who are serving a prison sentence or undergoing forensic psychiatric treatment. The specific research questions are as follows:

- Does pharmacological treatment reduce criminal recidivism? (primary outcome)
- Does pharmacological treatment reduce ADHD symptoms? (secondary outcome)
- Does pharmacological treatment affect rehabilitation‐related factors (including norm‐breaking/antisocial behavior, adaptation to society, well‐being, and global function)? (secondary outcome)

## 2 Materials and Methods

The study was conducted in accordance with the recommendations of the PRISMA 2020 Statement (Page et al. 2021) and the AMSTAR requirements (Shea et al. 2007). This review was not preregistered.

### 2.1 Database Search Methodology

A systematic search for relevant literature was conducted using the PubMed database. We applied the following search terms: tiab (“hyperkinetic disorder*” OR “attention‐deficit hyperactivity disorder*” OR “attention‐deficit/hyperactivity‐disorder*” OR “attention‐deficit/hyperactivity disorder*” OR “attention‐deficit hyperactivity‐disorder*” OR “attention‐deficit hyperactivity disorder*” OR ADHD) AND tiab (violen* OR criminal* OR prison* OR forensic* OR offen* OR convict* OR sentence*) AND tiab (*methylphenidate* OR dexamphetamine OR lisdexamphetamine OR *amphetamine* OR atomoxetine OR guanfacine OR clonidine OR modafinil). The database search was performed on September 6, 2023. No constraints were used when searching the literature.

### 2.2 Study Selection

Two reviewers (M. R. and A. L. B.) independently screened titles and abstracts for inclusion. Full‐text studies were obtained for publications deemed potentially relevant by at least one reviewer. Three independent reviewers screened full‐text studies against the predetermined inclusion criteria (A.C., M.R., and A.L.B.).

### 2.3 Inclusion Criteria

### Inclusion criteria are as follows

- original research studies published in English‐language peer‐reviewed scientific journals following the IMRaD format (Mogull 2017);
- excluding parallel publications and published governmental official reports;
- participants meeting the criteria for ADHD diagnosis according to any DSM version;
- participants incarcerated at the start of recommended pharmacological treatment for ADHD as stated by Faraone et al. (2021);
- evaluating the effects of pharmacological treatment (metylphenidate, which is a central stimulant) on either criminal recidivism, ADHD symptoms, global function, norm‐breaking/antisocial behavior, adaptation to society/institutional behavior, cognitive function, and well‐being.

### 2.4 Primary Outcome Measure

The primary outcome, criminal recidivism, refers to a relapse into the criminal behavior after receiving sanctions or undergoing intervention for a previous offense. Notably, there is no universally accepted specific definition of criminal recidivism (Zgoba and Salerno 2017). We define criminal recidivism, in accordance with the National Institute of Justice, as criminal acts that result in rearrest, reconviction, or return to prison with or without a new sentence during 3 years following the person's release (https://nij.ojp.gov/topics/corrections/recidivism).

### 2.5 Secondary Outcome Measures

The secondary outcome measures were ADHD symptoms, global function, norm‐breaking/antisocial behavior, adaptation to society/institutional behavior, cognitive function, and well‐being. In assessing ADHD severity, we only considered questionnaires specifically designed to evaluate ADHD‐related symptoms, as determined by either self‐report or assessment by a clinician. Furthermore, the rehabilitation‐related secondary outcomes of norm‐breaking behavior and adaptation to society may be largely dependent on different contexts and definitions, we therefore arbitrarily included related measures that were deemed relevant from each study. Antisocial behaviors are often defined as actions that violate the basic rights of others by committing crimes or nuisances (Calkins and Keane 2009). Global functioning is measured by clinicians who rate the social, occupational, and psychological functioning of an individual by different global functioning tests such as global assessment of functioning (GAF; first described in the DSM‐IV‐TR on page 34) and Clinical Global Impressions (CGI) scale (Busner and Targum 2007).

### 2.6 Data Extraction

Data (study characteristics; participants’ characteristics; pharmacological treatment; measured outcome results) were independently extracted and documented by three authors (A.C., M.R., and A.L.B.) on an Excel sheet with pre‐specified variables. In case of disagreement, the authors jointly reviewed the study to reach a consensus. Extracted study characteristics were sample size, sample characteristics, study design, length of intervention, drug and dosage used, mode of administration, control treatment, illegal drug control, start of treatment in relation to release from prison, psychosocial interventions, incarceration setting, drop‐out level, country of study, inclusion criteria, and exclusion criteria. Extracted patient characteristics were age, gender, educational level, criminal characteristics, comorbidity, and cognitive/intellectual functioning. Extracted outcome variables were criminal recidivism, ADHD symptoms (both clinician‐ and self‐rated), rehabilitation‐related factors in terms of global function, norm‐breaking/antisocial behavior, adaptation to society/institutional behavior, cognitive function, and well‐being.

### 2.7 Risk of Bias Assessment

We assessed the risk of bias using the Cochrane Risk of Biases (ROB 2) tool (Sterne et al. 2019), a tool that renders the risk of bias (low risk of bias/some concerns/high risk of bias) in randomized trials across five domains: 1) randomization process, (2) deviations from the assigned interventions, (3) missing outcome data, (4) measurement of the outcome, and (5) selection of the reported results. We only evaluated bias for the outcome of ADHD symptoms in the three RCT studies (Asherson et al. 2023; Ginsberg and Lindefors 2012; Konstenius et al. 2014), and not the two follow‐up studies (Ginsberg et al. 2012, 2015) based on the RCT of Ginsberg and Lindefors (2012). The ROB 2 ratings are reported in Tables S1 and S5.

## 3 Results

### 3.1 Literature Search

**FIGURE 1:**
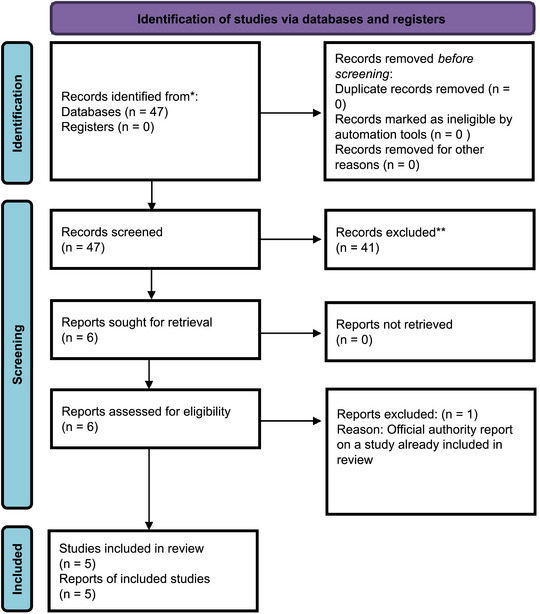
PRISMA 2020 flow diagram for new systematic reviews which included searches of databases and registers only. *Consider, if feasible to do so, reporting the number of records identified from each database or register searched (rather than the total number across all databases/registers). **If automation tools were used, indicate how many records were excluded by a human and how many were excluded by automation tools.

The literature search generated 47 records (PubMed). No abstracts were excluded because of duplicates. The abstracts were extracted and screened, leading to the exclusion of 41 abstracts for not meeting the inclusion criteria. Finally, six full‐text studies were screened and assessed for inclusion. One study was excluded since it was published in parallel as an official report from a governmental agency (see Asherson et al. 2022). Five studies were included in this systematic review. See Figure 1 for the PRISMA flow diagram.

### 3.2 Study and Patient Characteristics

A total of five studies published between 2012 and 2023 were included in this systematic review (Asherson et al. 2023; Ginsberg et al. 2012, 2015; Ginsberg and Lindefors 2012; Konstenius et al. 2014). The Ginsberg studies (Ginsberg et al. 2012, 2015; Ginsberg and Lindefors 2012) were based on the same prison cohort. This systematic review includes a total of 284 inmates (*n* = 30, 54 and 200). All five studies were based on randomized, double‐blind, placebo‐controlled trials. However, two of the five studies were randomized, double‐blind, placebo‐controlled trials followed by an open‐label extension (Ginsberg et al. 2012; Ginsberg and Lindefors 2012) and one of the five studies was a one, and 3 years, naturalistic follow‐up study of the RCT (Ginsberg et al. 2015). Four studies included inmates from Sweden (Ginsberg et al. 2012, 2015; Ginsberg and Lindefors 2012; Konstenius et al. 2014) and one study included inmates from UK (Asherson et al. 2023). Age was not consistently reported but it is estimated that most participants fe1ll within the age span of 20–40 years (range 16–65 years). All five studies were based on male inmates. The prison cohorts differed between studies regarding criminality characteristics, where the incarceration setting ranged from high‐security prison to young offender institutions. Most offenses involved violent or drug‐related crimes. See Table S1 for study characteristics and Table S2 for participants’ characteristics.

### 3.3 Risk of Bias Assessment

Based on the ROB 2 assessment, out of the three RCT studies evaluated, one study (Ginsberg and Lindefors 2012) was identified to have a low risk of bias, while another study (Asherson et al. 2023) demonstrated some concerns, and the third study (Konstenius et al. 2014) was judged to have a high risk of bias. Detailed judgments of domains and comments are reported in Tables S1 and S5.

### 3.4 Pharmacological Treatment

The five included RCT studies were based on three cohorts: Cohort 1 (Ginsberg et al. 2012, 2015; Ginsberg and Lindefors 2012), Cohort 2 (Asherson et al. 2023), and Cohort 3 (Konstenius et al. 2014). Osmotic release oral system (OROS) methylphenidate (Anatomical Therapeutic Chemical [ATC] classification system code N06BA04) was used in all studies, in different doses, uptitration schedules, and length of treatment.

The first cohort (Ginsberg et al. 2012, 2015; Ginsberg and Lindefors 2012) was designed as an RCT with open‐label extension during which methylphenidate was titrated from 36 mg/day, but not exceeding 1.3 mg/kg per day, to a maximum dosage of 72 mg/day for the four remaining weeks of the 5‐week RCT. The long‐term follow up dictate that at the 1‐year follow‐up, 20 (83%) of the 24 participants remained on ADHD medication (mean daily methylphenidate dose = 148 mg), and at the 3‐year follow‐up, 15 (75%) of the 20 participants were medicated for ADHD (mean daily methylphenidate dose = 144 mg) at the time. As part of the prison routines, participants were provided educational activities and treatment programs, in addition to their prescribed medication.

The second cohort (Asherson et al. 2023) uptitrated OROS methylphenidate doses from 18 mg/day to a maximum of 72 mg/day during 4 weeks with weekly increases with a mean final dose of 53.8 mg/day. The treatment duration was 8 weeks. As part of the prison routines, participants were provided educational activities and treatment programs, in addition to the study medication.

The third cohort (Konstenius et al. 2014) uptitrated OROS methylphenidate doses from 18 mg/day to a maximum dose of 180 mg/day during 19 days with an out‐clinic follow‐up. The treatment duration was 24 weeks. See Table S3 for pharmacological and non‐pharmacological interventions, such as cognitive behavioral therapy.

### 3.5 Criminal Recidivism

The effect of pharmacological ADHD treatment on criminal recidivism was only reported by Ginsberg et al. (2015). In this study, 25 ADHD‐diagnosed inmates who completed the 5‐week RCT (+47 weeks open‐label extension) were prospectively followed up after 1 year (*n* = 24) and 3 years (*n* = 20) after the end of the RCT and open‐label extension. The design of the self‐reported questionnaire addressing reoffending was not clearly described in the study. Regarding re‐offending after being released from prison among the inmates who had received treatment, at the 1‐year follow‐up, *n* = 3 (27.3%, 3/11) reported re‐offending, while at the 3 year follow‐up, *n* = 5 (33.3%, 5/15) reported reoffending. These results indicate a substantially lower relapse rate than expected based on the general population, which is between 70% and 80% according to The Swedish National Council for Crime Prevention as reported by Ginsberg et al. (2015). The self‐reported re‐offenses within the group who had received medical treatment included violent offenses (illegal threat or assault), driving without a license, illegal drug use, and fraud. See Table S4 for used outcome measures and Table 1 for main results.

### 3.6 ADHD Symptoms

The Ginsberg studies (Ginsberg et al. 2012, 2015; Ginsberg and Lindefors 2012) reported that methylphenidate effectively reduces ADHD symptoms. Specifically, Ginsberg et al. (2015) reported in their follow‐up study consistently lower levels of ADHD symptoms over time among the participants who continued their medication compared to the patients who had discontinued their medication (i.e., not compared to the placebo group). Furthermore, improvements in the methylphenidate group included enhanced verbal and visuospatial working memory, verbal reasoning, cognition‐related measures (continuous performance test), and reduced motor activity, with most cognitive gains appearing in the first 4 months and persisting for about a year (Ginsberg et al. 2012). Similarly, Konstenius et al. (2014) reported significant reductions in both self‐reported ADHD symptoms and clinician‐rated severity with methylphenidate treatment. In contrast, Asherson et al. (2023) observed no significant reductions in ADHD symptoms over 8 weeks, although both the treatment and placebo groups experienced a general symptom decrease. See Table S4 for used outcome measures and Table 1 for main results.

### 3.7 Rehabilitation‐Related Factors

The included studies present additional outcomes like the level of function, norm‐breaking behavior, adaptation to society, and well‐being. Asherson et al. (2023) observed no significant differences between the experimental group and the control group across a range of outcomes. In contrast, the Ginsberg studies (Ginsberg et al. 2012, 2015; Ginsberg and Lindefors 2012) consistently found improved global function in the experimental group. Konstenius et al. (2014) similarly reported improved global function in those pharmacologically treated group compared to placebo. Furthermore, Ginsberg et al. (2015) observed that self‐reported drug and alcohol use was below the cutoffs for problematic substance use at the 1‐year and 3‐year follow‐up, and Konstenius et al. (2014) found that methylphenidate significantly lowered drug relapse rate and increased treatment retention compared to placebo. See Table S4 for the used outcome measures and Table 1 for main results.

**TABLE 1:** Summary of main results.

	Outcomes								
	Criminal recidivism			ADHD symptoms			Rehabilitation‐related factors		
Study	Yes/No^a^	Measure	Effect size	Yes/No^b^	Measure	Effect size	Yes/No^c^	Measure	Effect size
Asherson et al. 2023	NA	NA	NA	No	CAARS‐O	0.06^d^	No	MEWS	0.16^d^
							No	WRAADDS	0.15^d^
							No	ARI‐S	−0.09^d^
							No	CORE‐OM	−0.01^d^
							No	BSI	0.07^d^
							No	MVQ	0.11^d^
							No	CGI	NA
							No	MOAS‐P	0.57^f^
							No	BRC‐P	0.95^g^
							No	Critical incidents	0.75^g^
							No	Education sessions	0.98^g^
Ginsberg and Lindefors 2012	NA	NA	NA	Yes	CAARS‐O: SV	**2.17** ^d^	Yes	GAF	**1.62** ^d^
				Yes	ASRS	**1.67** ^d^	Yes	CGI‐S	**2.36** ^d^
Ginsberg et al. 2012	NA	NA	NA	Yes	CAARS‐O: SV	**2.17** ^d^	Yes	GAF	**NA**
					ASRS	**NA**	Yes	CGI‐S	**NA**
Ginsberg et al. 2015^h^	Yes	Self‐report	NA	Yes	CAARS‐O: SV	1‐y: **0.55** ^e^ 3‐y: **0.66** ^e^	Yes	CGI‐S	1‐y: 0.50^e^ 3‐y: **0.68** ^e^
				Yes	ASRS	1‐y: **0.59** ^e^ 3‐y: **0.62** ^e^	No	GAF	1‐y: 0.35^e^ 3‐y: 0.37^e^
							No	QOLI	1‐y: 0.13^e^ 3‐y: 0.06^e^
							Yes	AUDIT	1‐y: **0.48** ^e^ 3‐y: **0.54** ^e^
							Yes	DUDIT	1‐y: **0.56e** 3‐y: **0.53** ^e^
Konstenius et al. 2014	NA	NA	NA	Yes	CAARS: SV	NA	Yes	CGI	NA
							NA	OQ45	NA
							Yes	Urine toxicology	**NA**

*Note*: Bolded = significant at *p* < 0.05.

^a^ Does pharmacological treatment reduce criminal recidivism?

^b^ Does pharmacological treatment reduce ADHD symptoms?

^c^ Does pharmacological treatment affect rehabilitation‐related factors?

^d^ Cohen's *d*.

^e^ Eta squared (*η* ^2^).

^f^ Estimated OR (odds ratio).

^g^ Estimated IRR (incidence rate ratio).

^h^ Medicated versus non‐medicated.

## 4 Discussion

The connection between ADHD and criminality is complex. This systematic review encompasses five studies (Asherson et al. 2023; Ginsberg et al. 2012, 2015; Ginsberg and Lindefors 2012; Konstenius et al. 2014), consisting of three randomized control trials and two long‐term follow‐up studies from one of the three randomized control trials conducted in different prison settings. Although the empirical results of our specific research question are scarce—criminal recidivism was only reported in one study—the gathered scientific evidence could indicate a potential effect of pharmacological ADHD treatment on reducing criminal recidivism—possibly through alleviating ADHD symptoms and improving rehabilitation. Although our findings should not be considered conclusive or generalizable to all prison populations or sub‐populations, the overall results highlight the need for more research on how pharmacological treatment/medication for ADHD can influence criminal behavior.

### 4.1 Criminal Recidivism (Primary Outcome)

It has been suggested that pharmacological ADHD treatment is associated with a lower risk of criminal behavior and criminal recidivism among individuals with ADHD (Faraone et al. 2021; Lichtenstein et al. 2012; Young et al. 2018). In our systematic review, there was only one study that specifically reported criminal recidivism (Ginsberg et al. 2015). That study included 25 participants who were prospectively followed, and during the 1‐year follow‐up, 24 of the initial participants were reassessed, revealing that three individuals out of 11 released from prison (amounting to 27.3%) reported criminal reoffending. More strikingly, at the 3‐year follow‐up, the participant count was reduced to 20, out of whom five of 15 released from prison (equating to 33.3%) reported instances of reoffending. The uncertainty related to these figures is, however, important to note since the outcome was based on *self‐reported* criminal recidivism. Additionally, the study's findings must be considered with caution due to its small participant number. Furthermore, the study compares these obtained results with the expected relapse rates of formerly imprisoned individuals, which are approximately 70%–80% according to official numbers from The Swedish National Council for Crime Prevention (Ginsberg et al. 2015). These rates include both arrests and suspicions. In addition, there was no untreated control group in the prospective follow‐up studies, meaning that their study should be considered a naturalistic follow‐up, which inherently lacks the robustness of randomized controlled trials. These weaknesses should be incorporated when evaluating the evidence gathered.

We find the lack of published studies examining the effect of pharmacological ADHD treatment on criminal recidivism in inmates diagnosed with ADHD surprising, considering the large number of incarcerated persons who report ADHD symptoms. Additionally, we posit that the financial burden jointly with other related costs for societies that can be linked to criminal behavior and criminal recidivism is substantial, which makes the topic urgent to pursue if there is a chance of lower relapse rates among persons with ADHD who receives medication. Based on registry data, it has been reported that pharmacological ADHD treatment is associated with a lower criminality rate (Lichtenstein et al. 2012), which is consistent with expert consensus on the conceptual idea of how criminality, to some degree, may be hampered by medication (Young et al. 2018). To reiterate, the lack of published RCT‐trials examining the effects of pharmacological ADHD treatment is surprising since several sources, as referenced above, seem to suggest that pharmacological treatment ought to lead to a decrease in criminal recidivism.

### 4.2 ADHD Symptoms (Secondary Outcome)

Asherson et al. (2023) found no significant effects of methylphenidate versus placebo on ADHD symptoms among inmates, in contrast to the other studies in this review. However, this finding may be questionable since treatment adherence was low (roughly half of prescribed medication taken) compared to close to 100% adherence reported by Ginsberg and Lindefors (2012). Drop‐out was high in Konstenius et al. (2014) but they managed this inconsistency by defining study completion as having received at least 75% of the study medication. Since no drug screening was performed throughout the study, in contrast with Konstenius et al. (2014) and Ginsberg and Lindefors (2012), concurrent use of illicit drugs could have affected treatment response. The relatively low dose of methylphenidate is also a possible explanation for failure to respond. In sum, within‐group adherence to treatments, selection bias, and different treatment regimens may all contribute to differences in treatment effectiveness.

### 4.3 Rehabilitation‐Related Factors (Secondary Outcomes)

The range of rehabilitation‐related factors reported by the included studies is diverse in specificity and outcome. However, the pattern is largely like the findings regarding the effects of pharmacological ADHD treatment. Based on a comprehensive list of rehabilitation‐related factors, Asherson et al. (2023) observed no significant effects, in contrast to the Ginsberg studies (Ginsberg et al. 2012, 2015; Ginsberg and Lindefors 2012) and Konstenius et al. (2014) who consistently found improved global function from treatment. More importantly, Konstenius et al. (2014) found that methylphenidate significantly lowered drug and alcohol use. ADHD and SUD are associated, and it is estimated that 20%–50% of adults with SUD also suffer from ADHD (Gordon, Tulak, and Troncale 2004), and conversely, 15%–50% of adults with ADHD suffer from SUD (Sullivan and Rudnik‐Levin 2001). Medication for ADHD has also shown a positive effect on SUD, but the effects largely seem to be dependent on dosage and whether the person also has other kinds of psychiatric co‐morbidity (Levin et al. 2015).

The included studies in this review vary substantially in terms of dosage, where Konstenius et al. (2014) administered the highest doses, and Ginsberg and Lindefors (2012) administered a dosage that was higher than Asherson et al. (2023). Konstenius et al. (2014) argued that one possible explanation for the less‐than‐optimal results in previous studies of individuals with ADHD and concurrent SUD might have been due to the administration of too low doses.

Differences in rehabilitation‐related factors between the included studies may also stem from the levels of participant compliance. This implies that the differences in the actual dose taken, rather than the dose prescribed, may partly explain the different outcomes. Furthermore, there are indications that pharmacological ADHD treatment may alleviate SUD (cocaine use disorder) above and beyond improving ADHD symptoms (Levin et al. 2015), which may cloud the internal validity of the treatment since ADHD and SUD are likely to be independently linked to criminal behavior and success in rehabilitation. In the included studies in this review, all participants in Ginsberg and Lindefors's (2012) study had a lifetime history of SUD, and in Asherson et al.'s (2023) study, 74% of the participants had problematic alcohol use and 97% of the participants had illicit drug use. This underlines the importance of considering actual medication intake, rather than dosage alone, when assessing treatment efficacy.

### 4.4 Clinical and Societal Relevance

The overall findings from this systematic review are not conclusive, but we posit that there are clinical implications worth considering. The societal implications of how criminal recidivism possibly may be decreased by common pharmacological ADHD treatment are substantial. We advocate that this preliminary finding and potential mechanism should be investigated further since criminality is a heavy financial burden for society at large and a long‐term individual suffering. Bringing attention and awareness of the prevalence of ADHD, as well as the high level of psychiatric comorbidity among prison populations, and exploring the extent of ADHD medication as a beneficial treatment option may reduce stigma and misconceptions of incarcerated individuals, which in turn could facilitate rehabilitation further.

### 4.5 Limitations

Systematic reviews are inherently vulnerable to the quality of the included studies, and this systematic review is no exception. The included studies exhibit significant variability in terms of participant age, ADHD symptom severity, method of ADHD‐symptom assessment, psychiatric comorbidity, dosage, adherence, and treatment procedures. This variability makes it difficult to interpret our findings universally applicable across different prison populations.

Perhaps the most crucial limitation for a balanced understanding of our findings is the small number of published RCT studies investigating the effects of pharmacological treatment for ADHD on criminal recidivism, ADHD symptoms, and rehabilitation in persons diagnosed with ADHD who are serving a prison sentence or undergoing forensic psychiatric treatment. This is in part due to the narrow scope of this systematic review. Other outcomes, such as aggression, would likely have rendered more published results. Our systematic search resulted in the finding of only one RCT that investigated the efficacy of pharmacological ADHD treatment on criminal recidivism in inmates diagnosed with ADHD. It is important to note that even if we claim evidence of absence for studies to answer the research question, this is based on the stated inclusion criteria and search strategy. This systematic review relies on published peer‐reviewed scientific studies written in English and indexed in the PubMed database. So‐called “gray literature” (reports, theses, working papers, conference papers, etc.) was intendedly not searched for, because the added value of including gray literature was not considered to outweigh the net cost in time and resources required to conduct such a search (e.g., Paez 2017). Furthermore, this systematic review only included one pharmacological ADHD treatment (methylphenidate, which is a central stimulant), which could affect the generalizability compared to other medications. There is also a risk that different medications could interact to varying degrees in relation to often supplemented non‐medical rehabilitation‐related factors such as education, cognitive behavioral therapy (CBT), and training.

The findings highlight the lack of research on the effectiveness of pharmacological treatment for reducing criminal reoffending, managing ADHD symptoms, and aiding rehabilitation in inmates with ADHD. This stresses the need for more targeted research in this crucial societal and clinical area. It is, however, important to consider the abovementioned limitations when establishing the results.

## 5 Conclusion

We conclude that there is limited empirical evidence to support the efficacy of pharmacological ADHD treatment on criminal recidivism in inmates diagnosed with ADHD. Still, there are indications to suggest that these treatments can reduce ADHD symptoms and enhance rehabilitation outcomes which may, in turn, lower the rate of reoffending. More research is needed to identify how several other important factors interact with the efficacy of pharmacological treatment on ADHD symptoms to provide meaningful and realistic therapeutic success. We point to the novelty and current scarcity of published research in this area, highlighting the urgent need for more targeted and in‐depth investigations.

## Author Contributions

**A. Carlander**: conceptualization, data curation, formal analysis, investigation, methodology, project administration, supervision, validation, visualization, writing–original draft, writing–review and editing. **M. Rydell**: conceptualization, data curation, formal analysis, investigation, methodology, validation, visualization, writing–original draft, writing–review and editing. **H. Kataoka**: data curation, formal analysis, investigation, validation, visualization, writing–original draft, writing–review and editing. **M. Hildebrand Karlén**: data curation, formal analysis, investigation, validation, visualization, writing–original draft, writing–review and editing. **A.‐S. Lindqvist Bagge**: conceptualization, data curation, formal analysis, investigation, methodology, project administration, supervision, validation, visualization, writing–original draft, writing–review and editing.

## Conflicts of Interest

The authors declare no conflicts of interest.

### Peer Review

The peer review history for this article is available at https://publons.com/publon/10.1002/brb3.70120.

## Supporting information



Supporting Information

## Data Availability

Data sharing is not applicable to this article as no datasets were generated or analyzed during the current study.

## References

[brb370120-bib-0001] American Psychiatric Association . 2013. Diagnostic and Statistical Manual of Mental Disorders (5th ed.). Washington, DC: American Psychiatric Publishing. 10.1176/appi.books.9780890425596.

[brb370120-bib-0002] Asherson, P. , J. Buitelaar , S. V. Faraone , and L. A. Rohde . 2016. “Adult Attention‐Deficit Hyperactivity Disorder: Key Conceptual Issues.” Lancet Psychiatry 3, no. 6: 568–578. 10.1016/S2215-0366(16)30032-3. 27183901

[brb370120-bib-0003] Asherson, P. , L. Johansson , R. Holland , et al. 2022. “OROS‐Methylphenidate to Reduce ADHD Symptoms in Male Prisoners Aged 16–25 Years: A RCT.” Efficacy and Mechanism Evaluation 9, no. 6. 10.3310/THEI8200. 35793424

[brb370120-bib-0004] Asherson, P. , L. Johansson , R. Holland , et al. 2023. “Randomised Controlled Trial of the Short‐Term Effects of Osmotic‐Release Oral System Methylphenidate on Symptoms and Behavioural Outcomes in Young Male Prisoners With Attention Deficit Hyperactivity Disorder: CIAO‐II Study.” British Journal of Psychiatry 222, no. 1: 7–17. 10.1192/bjp.2022.77. PMC7613969 35657651

[brb370120-bib-0005] Baggio, S. , A. Fructuoso , M. Guimaraes , et al. 2018. “Prevalence of Attention Deficit Hyperactivity Disorder in Detention Settings: A Systematic Review and Meta‐Analysis.” Front Psychiatry 9: 331. 10.3389/fpsyt.2018.00331. 30116206 PMC6084240

[brb370120-bib-0006] Bihlar Muld, B. , J. Jokinen , S. Bölte , and T. Hirvikoski . 2015. “Long‐Term Outcomes of Pharmacologically Treated Versus Non‐Treated Adults With ADHD and Substance Use Disorder: A Naturalistic Study.” Journal of Substance Abuse Treatment 51: 82–90. 10.1016/j.jsat.2014.11.005. 25491733

[brb370120-bib-0007] Busner, J. , and S. D. Targum . 2007. “The Clinical Global Impressions Scale: Applying a Research Tool in Clinical Practice.” Psychiatry (Edgmont) 4, no. 7: 28–37. https://www.ncbi.nlm.nih.gov/pubmed/20526405. PMC2880930 20526405

[brb370120-bib-0008] Calkins, S. D. , and S. P. Keane . 2009. “Developmental Origins of Early Antisocial Behavior.” Development and Psychopathology 21, no. 4: 1095–1109. 10.1017/S095457940999006X. 19825259 PMC2782636

[brb370120-bib-0009] Chang, Z. , P. Lichtenstein , N. Langstrom , H. Larsson , and S. Fazel . 2016. “Association Between Prescription of Major Psychotropic Medications and Violent Reoffending After Prison Release.” Jama 316, no. 17: 1798–1807. 10.1001/jama.2016.15380. 27802545 PMC5100822

[brb370120-bib-0010] Faraone, S. V. , T. Banaschewski , D. Coghill , et al. 2021. “The World Federation of ADHD International Consensus Statement: 208 Evidence‐Based Conclusions About the Disorder.” Neuroscience and Biobehavioral Reviews 128: 789–818. 10.1016/j.neubiorev.2021.01.022. 33549739 PMC8328933

[brb370120-bib-0011] Fayyad, J. , N. A. Sampson , I. Hwang , et al. 2017. “The Descriptive Epidemiology of DSM‐IV Adult ADHD in the World Health Organization World Mental Health Surveys.” Attention Deficit and Hyperactivity Disorders 9, no. 1: 47–65. 10.1007/s12402-016-0208-3. 27866355 PMC5325787

[brb370120-bib-0012] Ginsberg, Y. , T. Hirvikoski , M. Grann , and N. Lindefors . 2012. “Long‐Term Functional Outcome in Adult Prison Inmates With ADHD Receiving OROS‐Methylphenidate.” European Archives of Psychiatry and Clinical Neuroscience 262, no. 8: 705–724. 10.1007/s00406-012-0317-8. 22526730 PMC3491195

[brb370120-bib-0013] Ginsberg, Y. , N. Långström , H. Larsson , and N. Lindefors . 2015. “Long‐Term Treatment Outcome in Adult Male Prisoners With Attention‐Deficit/Hyperactivity Disorder.” Journal of Clinical Psychopharmacology 35, no. 5: 535–543. 10.1097/jcp.0000000000000395. 26284932

[brb370120-bib-0014] Ginsberg, Y. , and N. Lindefors . 2012. “Methylphenidate Treatment of Adult Male Prison Inmates With Attention‐Deficit Hyperactivity Disorder: Randomised Double‐Blind Placebo‐Controlled Trial With Open‐Label Extension.” British Journal of Psychiatry 200, no. 1: 68–73. 10.1192/bjp.bp.111.092940. 22075648

[brb370120-bib-0015] Gordon, S. M. , F. Tulak , and J. Troncale . 2004. “Prevalence and Characteristics of Adolescents Patients With Co‐Occurring ADHD and Substance Dependence.” Journal of Addictive Diseases 23, no. 4: 31–40. 10.1300/J069v23n04_03. 15339712

[brb370120-bib-0016] Konstenius, M. , N. Jayaram‐Lindström , J. Guterstam , O. Beck , B. Philips , and J. Franck . 2014. “Methylphenidate for Attention Deficit Hyperactivity Disorder and Drug Relapse in Criminal Offenders With Substance Dependence: A 24‐Week Randomized Placebo‐Controlled Trial.” Addiction 109, no. 3: 440–449. 10.1111/add.12369. 24118269 PMC4226329

[brb370120-bib-0017] Levin, F. R. , J. J. Mariani , S. Specker , et al. 2015. “Extended‐Release Mixed Amphetamine Salts vs Placebo for Comorbid Adult Attention‐Deficit/Hyperactivity Disorder and Cocaine Use Disorder.” JAMA Psychiatry 72, no. 6: 593. 10.1001/jamapsychiatry.2015.41. 25887096 PMC4456227

[brb370120-bib-0018] Lichtenstein, P. , L. Halldner , J. Zetterqvist , et al. 2012. “Medication for Attention Deficit–Hyperactivity Disorder and Criminality.” New England Journal of Medicine 367, no. 21: 2006–2014. 10.1056/nejmoa1203241. 23171097 PMC3664186

[brb370120-bib-0019] Mogull, S. A. 2017. Scientific and Medical Communication: A Guide for Effective Practice (3rd ed.). New York, NY: Routledge.

[brb370120-bib-0020] Mohr‐Jensen, C. , and H.‐C. Steinhausen . 2016. “A Meta‐Analysis and Systematic Review of the Risks Associated With Childhood Attention‐Deficit Hyperactivity Disorder on Long‐Term Outcome of Arrests, Convictions, and Incarcerations.” Clinical Psychology Review 48: 32–42. 10.1016/j.cpr.2016.05.002. 27390061

[brb370120-bib-0021] Moukhtarian, T. R. , R. E. Cooper , E. Vassos , P. Moran , and P. Asherson . 2017. “Effects of Stimulants and Atomoxetine on Emotional Lability in Adults: A Systematic Review and Meta‐Analysis.” European Psychiatry 44: 198–207. 10.1016/j.eurpsy.2017.05.021. 28646732

[brb370120-bib-0022] Paez, A. 2017. “Gray Literature: An Important Resource in Systematic Reviews.” Journal of Evidence Based Medicine 10, no. 3: 233–240. 10.1111/jebm.12266. 28857505

[brb370120-bib-0023] Page, M. J. , J. E. McKenzie , P. M. Bossuyt , et al. 2021. “The PRISMA 2020 Statement: An Updated Guideline for Reporting Systematic Reviews.” BMJ 372: n71. 10.1136/bmj.n71. 33782057 PMC8005924

[brb370120-bib-0024] Retz, W. , Y. Ginsberg , D. Turner , et al. 2021. “Attention‐Deficit/Hyperactivity Disorder (ADHD), Antisociality and Delinquent Behavior Over the Lifespan.” Neuroscience and Biobehavioral Reviews 120: 236–248. 10.1016/j.neubiorev.2020.11.025. 33271164

[brb370120-bib-0025] Retz, W. , and M. Rosler . 2010. “Association of ADHD With Reactive and Proactive Violent Behavior in a Forensic Population.” Attention Deficit and Hyperactivity Disorders 2, no. 4: 195–202. 10.1007/s12402-010-0037-8. 21432606

[brb370120-bib-0026] Shea, B. J. , J. M. Grimshaw , G. A. Wells , et al. 2007. “Development of AMSTAR: A Measurement Tool to Assess the Methodological Quality of Systematic Reviews.” BMC Medical Research Methodology 7: 10. 10.1186/1471-2288-7-10. 17302989 PMC1810543

[brb370120-bib-0027] Sterne, J. A. C. , J. Savovic , M. J. Page , et al. 2019. “RoB 2: A Revised Tool for Assessing Risk of Bias in Randomised Trials.” BMJ 366: l4898. 10.1136/bmj.l4898. 31462531

[brb370120-bib-0028] Sullivan, M. A. , and F. Rudnik‐Levin . 2001. “Attention Deficit/Hyperactivity Disorder and Substance Abuse. Diagnostic and Therapeutic Considerations.” Annals of the New York Academy of Sciences 931: 251–270. 10.1111/j.1749-6632.2001.tb05783.x. 11462745

[brb370120-bib-0029] Young, S. , G. Gudjonsson , P. Chitsabesan , et al. 2018. “Identification and Treatment of Offenders With Attention‐Deficit/Hyperactivity Disorder in the Prison Population: A Practical Approach Based Upon Expert Consensus.” BMC Psychiatry 18, no. 1: 281. 10.1186/s12888-018-1858-9. 30180832 PMC6122636

[brb370120-bib-0030] Young, S. , G. H. Gudjonsson , J. Wells , et al. 2009. “Attention Deficit Hyperactivity Disorder and Critical Incidents in a Scottish Prison Population.” Personality and Individual Differences 46, no. 3: 265–269. 10.1016/j.paid.2008.10.003.

[brb370120-bib-0031] Young, S. , O. Sedgwick , M. Fridman , et al. 2015. “Co‐Morbid Psychiatric Disorders Among Incarcerated ADHD Populations: A Meta‐Analysis.” Psychological Medicine 45, no. 12: 2499–2510. 10.1017/S0033291715000598. 25857258 PMC4531473

[brb370120-bib-0032] Zgoba, K. M. , and L. M. Salerno . 2017. “A Three‐Year Recidivism Analysis of State Correctional Releases.” Criminal Justice Studies 30, no. 4: 331–345. 10.1080/1478601x.2017.1364641.

